# Supplementary material for: Low-frequency vibrational modes in G-quadruplexes reveal the mechanical properties of nucleic acids
Source: Phys Chem Chem Phys. 2021 Jun 1;23(23):13250–60. doi: 10.1039/d0cp05404f (PMC8207511; doi:10.1039/d0cp05404f)
Supplement: CP-023-D0CP05404F-s001 [file CP-023-D0CP05404F-s001.pdf]

Electronic Supplementary Material (ESI) for Physical Chemistry Chemical Physics.
This journal is © the Owner Societies 2021

Supplementary Information for: Low-Frequency Vibrational Modes in G-Quadruplexes Reveal the Mechanical Properties of Nucleic Acids

Mario González-Jiménez, Gopakumar Ramakrishnan, Nikita V. Tukachev, Hans M. Senn, and Klaas Wynne*
School of Chemistry, University of Glasgow, Glasgow, G12 8QQ, UK

1. SUPPLEMENTARY FIGURES **2**
2. SUPPLEMENTARY TABLES **15**
3. SUPPLEMENTARY NOTES **17**

3.1. Supplementary Note 1: Water OKE spectra and subtraction procedure **17**
3.2. Supplementary Note 2: G4s melting thermodynamics **18**
3.3. Supplementary Note 3: Computational details **19**
3.3.1. Structure 19
3.3.2. Vibrational spectra 20
3.3.3. Structure of guanine complexes 22
3.3.4. OKE spectra 32
3.3.5. IR spectra 38
3.3.6. Normal modes active in OKE 39

4. SUPPLEMENTARY REFERENCES **43**

1. Supplementary figures

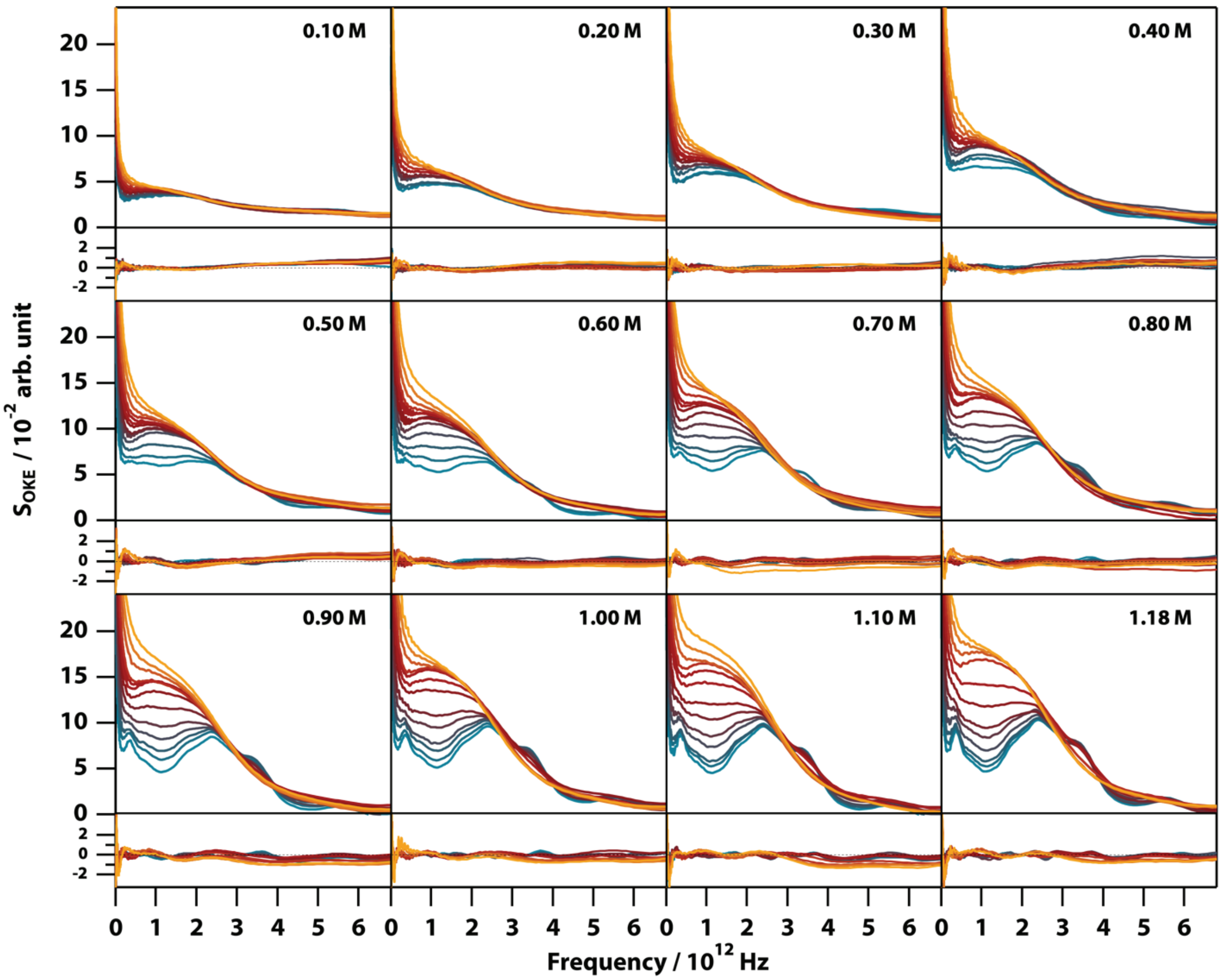

Figure S1. Optical Kerr-effect spectra of aqueous Na_2(5′-GMP) solutions at different concentrations and temperatures showing changes in the spectra due to the formation of stacks and G-quadruplexes. Temperatures run from 283 (blue) to 328 K (red) in steps of 5 K and from 328 K to 358 K (orange) in steps of 10 K. The fitting deviations (residuals) between each spectrum and the proposed model values are shown in the graphs below the spectra. (Linear scale version of Figure 2.)

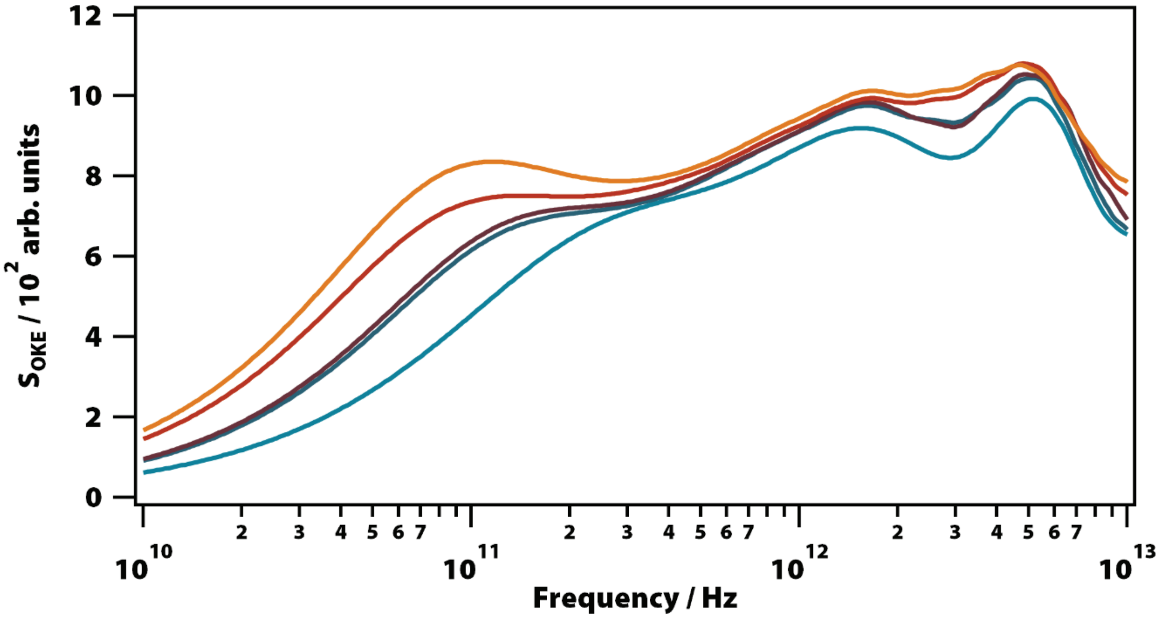

Figure S2. Experimental OKE spectra at 298 K of water (blue) and different NaCl solutions. The concentration of these solutions is: 0.5 M (dark blue), 1.0 M (purple), 2.0 M (red), and 4.0 M (yellow).

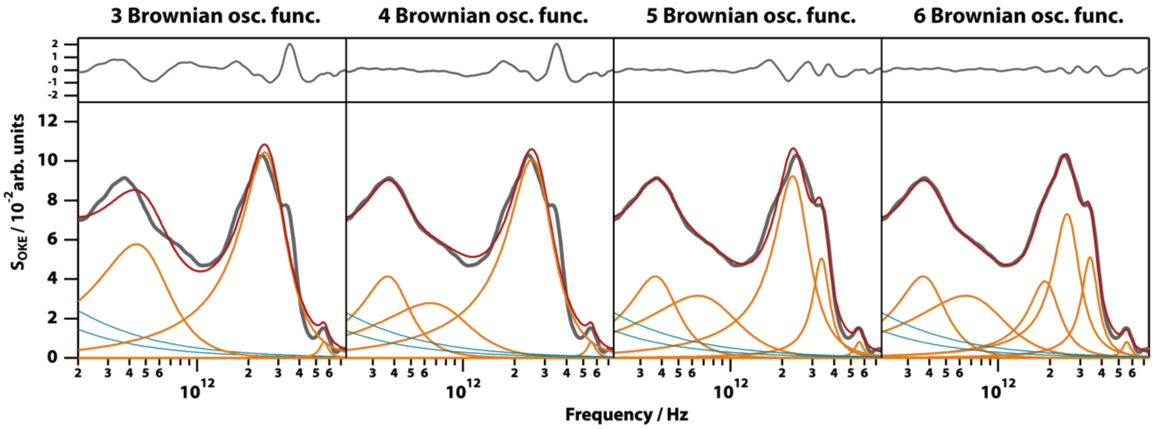

Figure S3. Experimental OKE spectrum at 283 K of a 1.18 M GMP aqueous solution (grey), fitted with 3, 4, 5 and 6 Brownian oscillator functions (yellow), keeping the diffusion associated functions constant (blue). Each graph shows the model with less standard deviation (red).

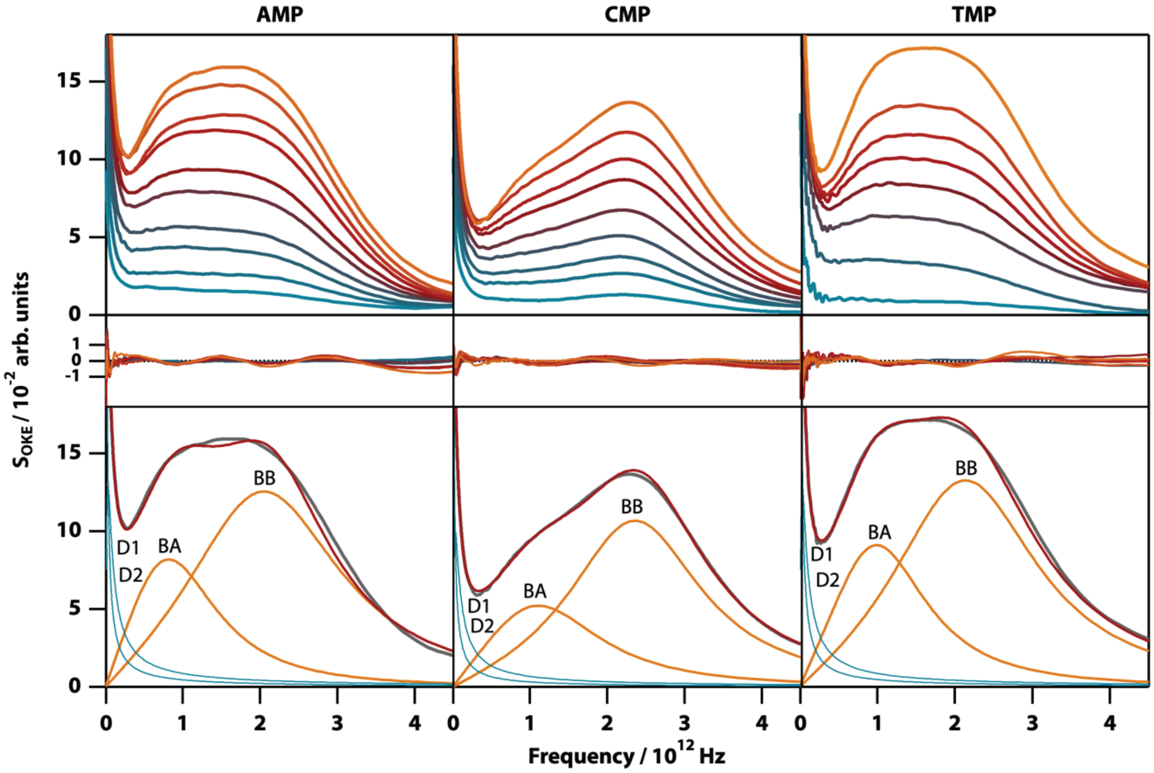

Figure S4. OKE spectra of aqueous solutions of three non-GMP nucleotides at 298 K demonstrating the fundamentally different form. Shown are the spectra for adenine (left column), cytosine (central), and thymidine monophosphate (right) at concentrations between 0.1 (blue) and 1.6 M (orange). Spectra are plotted in intervals of 0.1 M between 0.1 and 0.4 M and intervals of 0.2 M between 0.4 and 1.6 M for AMP and CMP and in intervals of 0.2 M for the whole concentration range of TMP (first row). The second row shows the fitting deviations (residuals) between each spectrum and the fittings and third row contains the 1.6 M spectrum for each nucleotide (grey), its fitting (red), and the functions used to model the data: Debye (blue) and Brownian oscillator (orange). (Linear scale version of Fig 3).

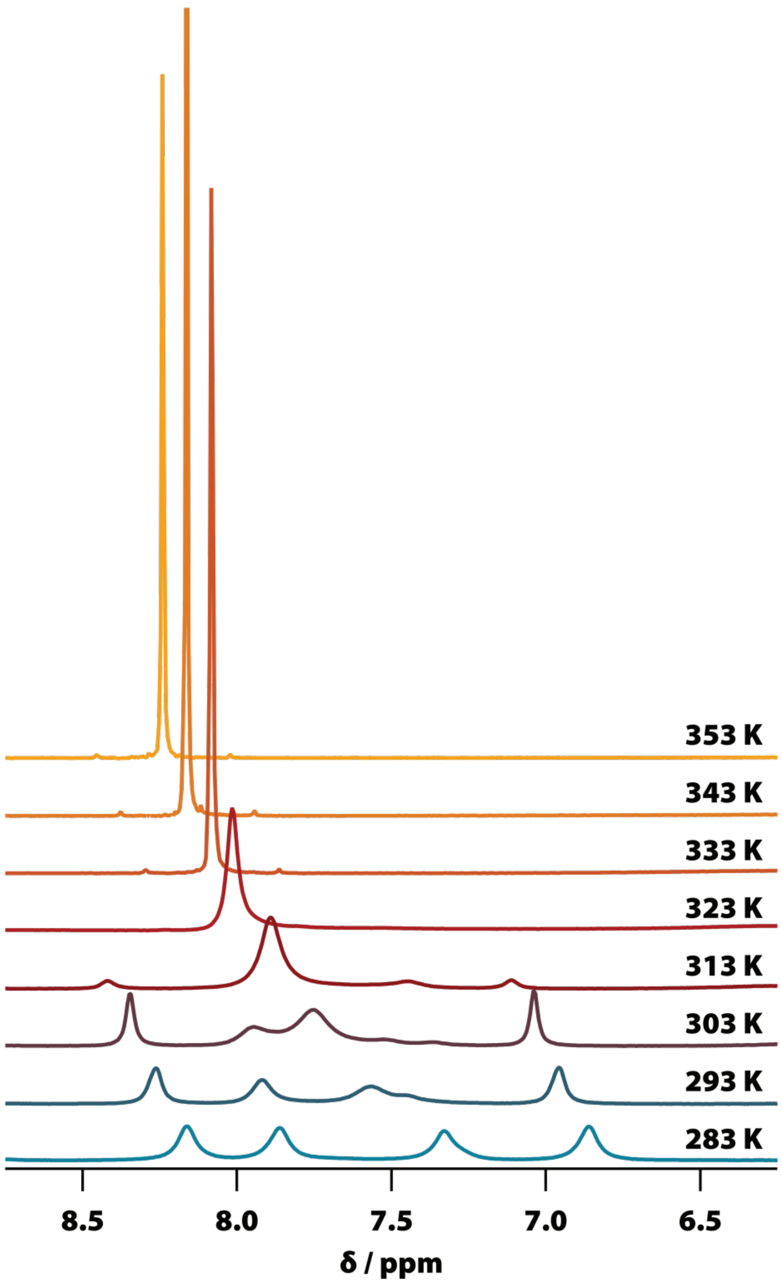

Figure S5. Influence of the temperature on the H8 region of the ^{1}H NMR spectra for Na_2(5′-GMP) 1.18 M .

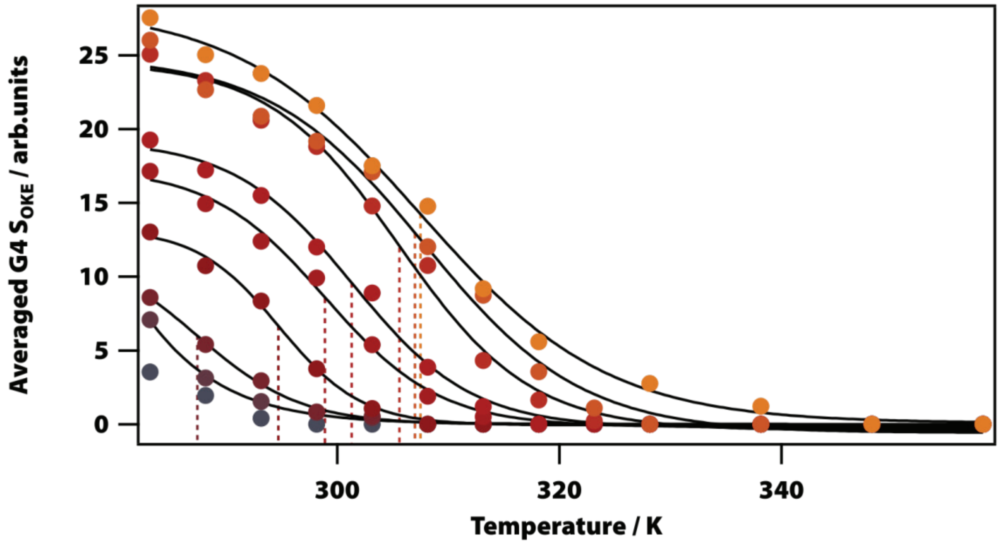

Figure S6. Melting curves of G4 conformation of Na2(5′-GMP) at different concentrations from 1.18 M (yellow) to 0.40 M (purple). Slashed lines represent the melting temperatures at each concentration. Results are shown in Supplementary Table 2.

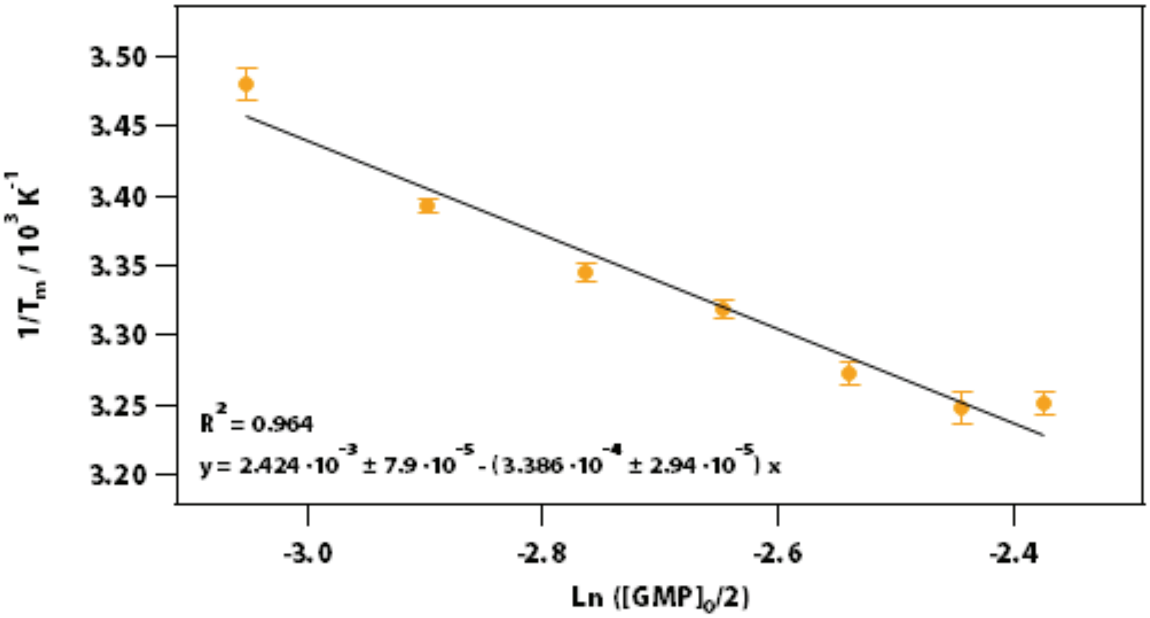

Figure S7. Determination of the standard-state van 't Hoff thermodynamic changes for the dissociation of the G-quadruplexes using the melting temperatures from Supplementary Figure 3. See also Supplementary Note 2.

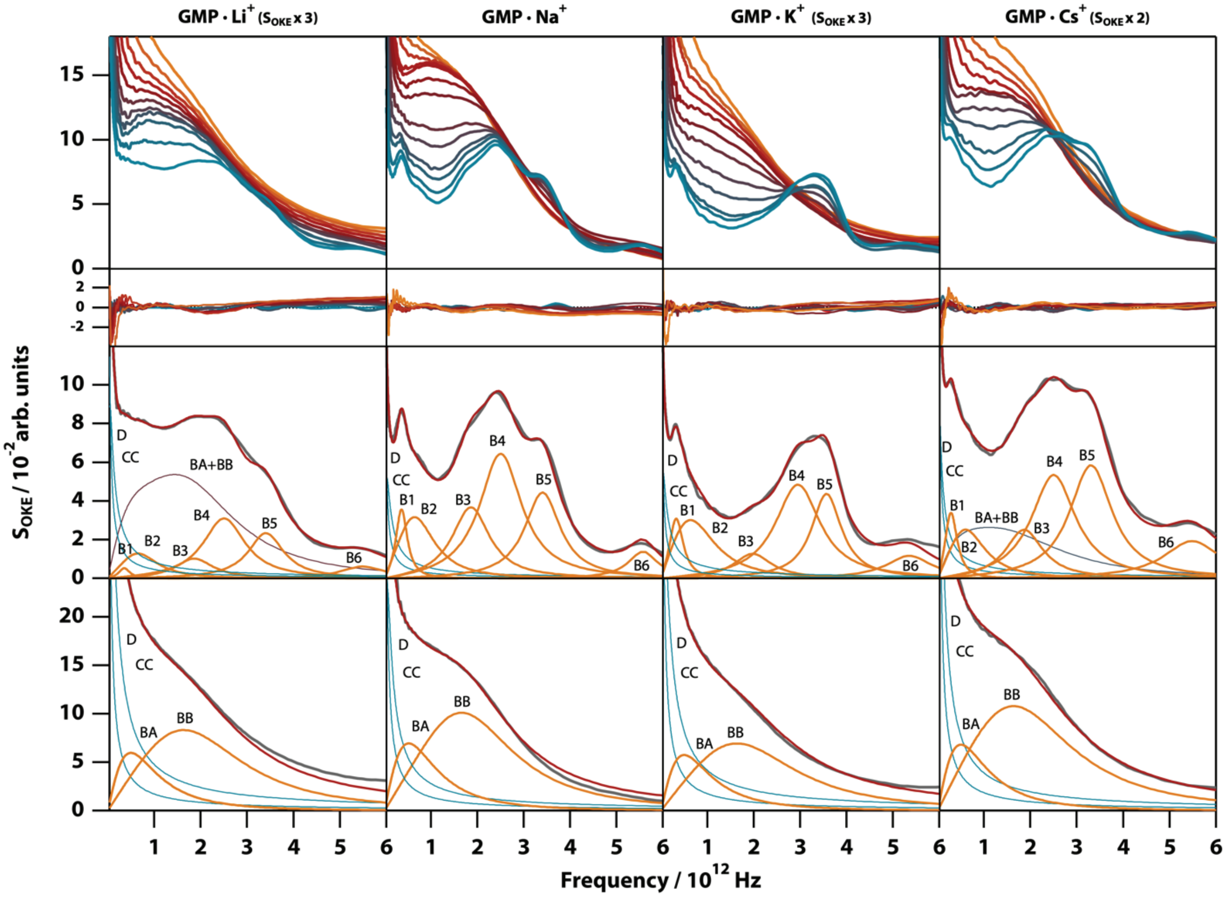

Figure S8. Temperature-dependent OKE spectra of GMP solutions with different cations. Top row, from left to right: OKE spectra of GMP in the presence of Li^+ (GMP 0.25 M, LiCl 1.0 M), Na^+ (Na_2(5'-GMP) 1.0 M), K^+ (GMP 0.2 M, KCl 0.5M), and Cs^+ solutions (GMP 0.4 M, CsCl 1.0 M). The temperature runs from 283 (blue) to 328 K (red) in steps of 5 K and from 328 K to 358 (orange) in steps of 10 K. Below the residuals between each spectrum and the fittings are shown. The remaining rows contain the spectrum for each case (grey) at 283 K (third row) and 358 K (fourth row) and the functions employed to fit the spectra. Diffusion associated functions (Debye and Cole-Cole) are coloured in red and vibration associated functions (Brownian oscillators) in orange. The blue band in the GMP·Li^+ and GMP·Cs^+ spectra at 283 K is the result of the sum of the two Brownian oscillators associated with GMP stack conformation (BA + BB). (Linear scale version of Figure 5).

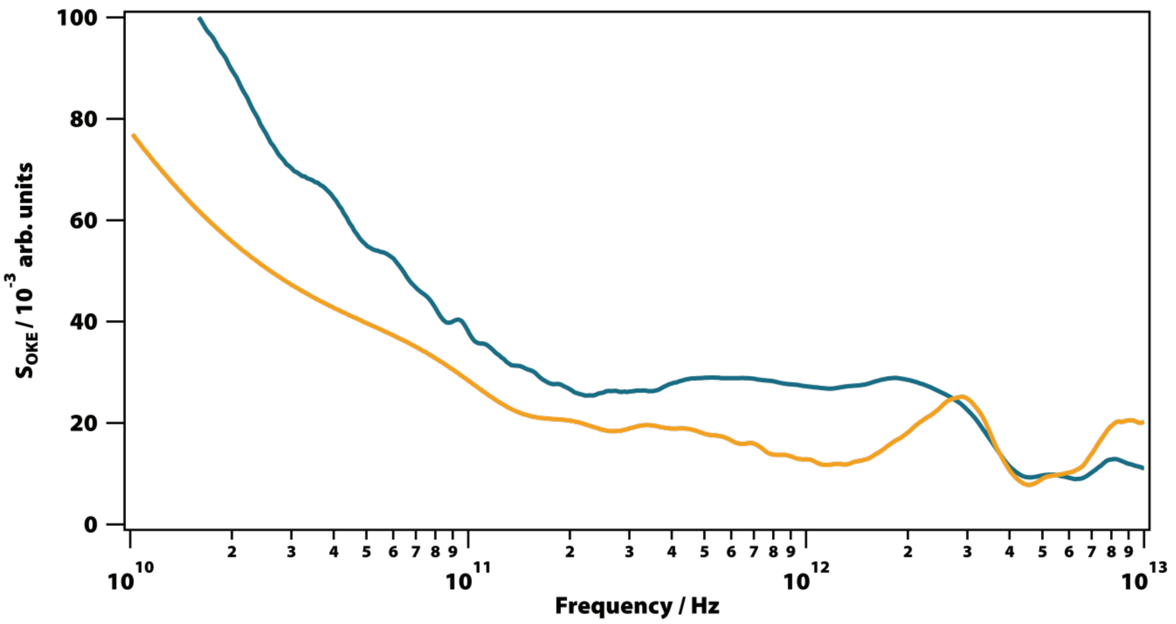

Figure S9. OKE spectrum of a solution of d($G_3CT_4G_3C$) 80 mg/ml (blue) and OKE spectrum of a solution of d($G_3CT_4G_3C$) 80 mg/ml and KCl 1.6 M (yellow). Both spectra were collected at 283 K.

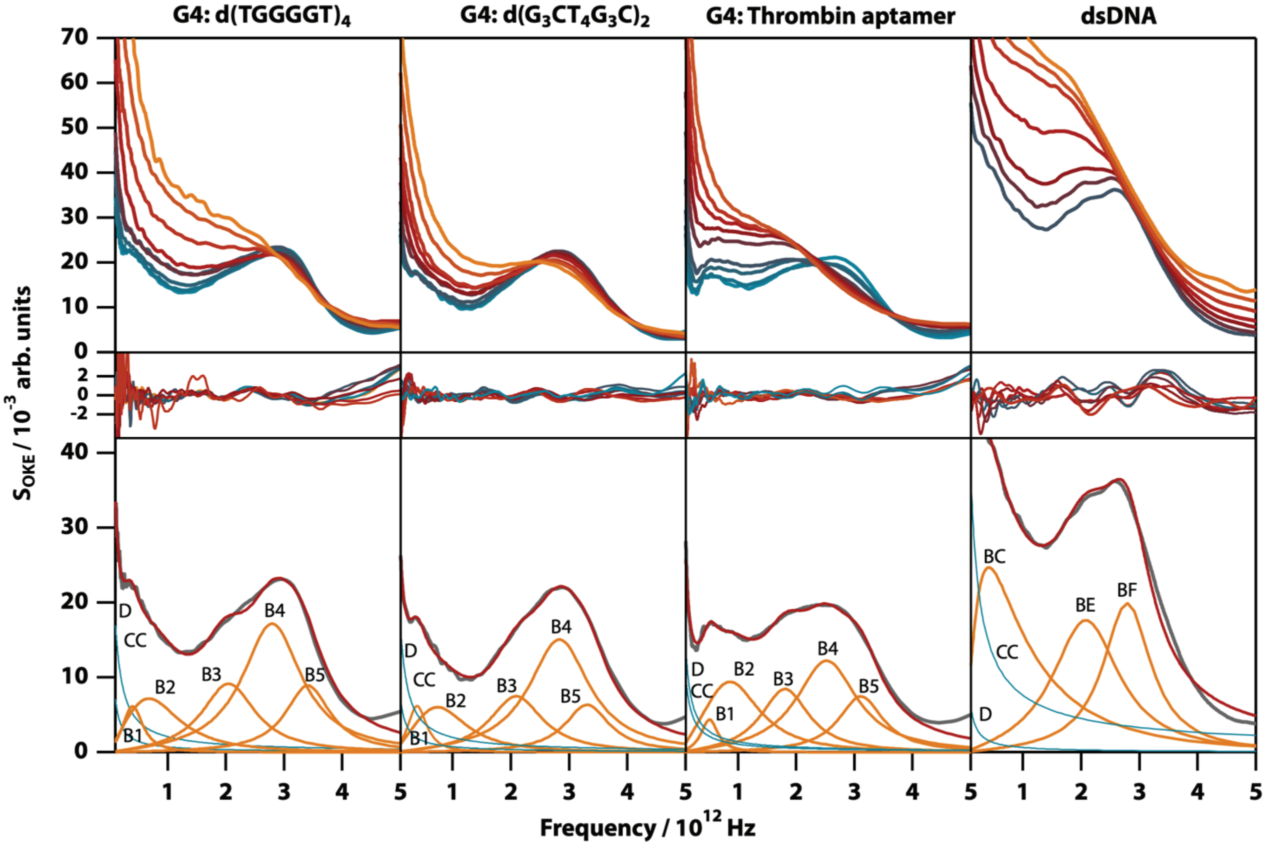

Figure S10. OKE spectra of solutions of three G4-forming oligomers and one double-helix forming oligomer demonstrating that the gigahertz-to-terahertz modes seen in GMP-based G-quadruplexes persist in oligomers. From left to right: Spectra of d(TGGGGT) (80 mg/ml, KCl 1.6 M), d(G3CT4G3C) (80 mg/ml, KCl 1.6 M), and TBA (80 mg/ml, KCl 0.4 M) between 283 (blue) to 318K (red) in steps of 5 K and from 318 K to 358 (orange) in steps of 10 K. Spectra of d(T2AT2A3TATAT3A2TA2)2 (right) between 298 (blue) and 358 K (orange) in intervals of 10 K. Residuals are shown below. The third row shows the spectrum of lower temperature for each oligomer (grey) and the functions used to model them. Debye and Cole-Cole functions are coloured in red and Brownian oscillators in orange. (Linear scale version of Fig 6).

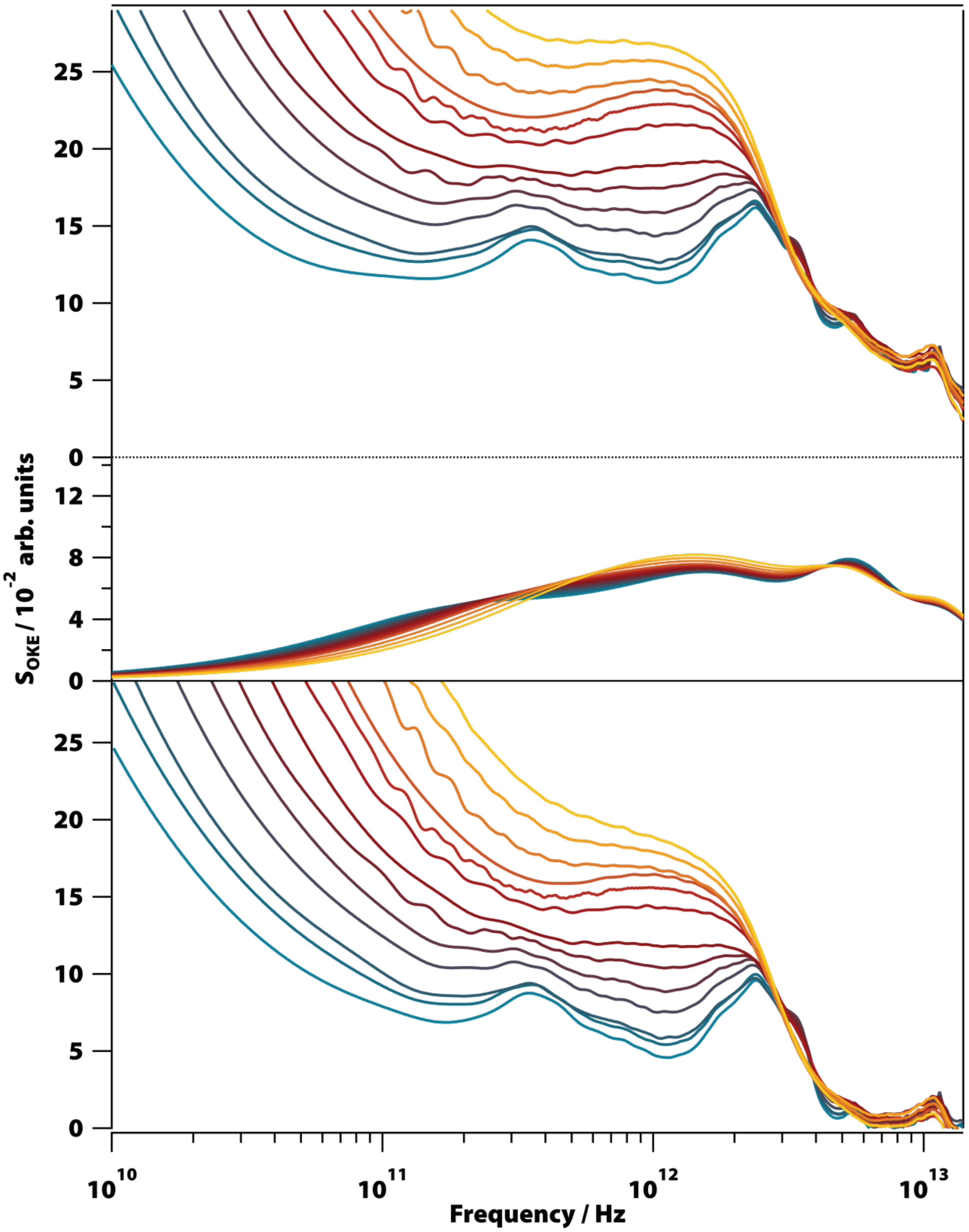

Figure S11. Example of solvent subtraction from OKE spectra (Na_2(5′-GMP) 1.10 M). To obtain the spectra of the solvated GMP (bottom) shown in Figure 1, water spectra (middle) were subtracted from the measured spectra (top).

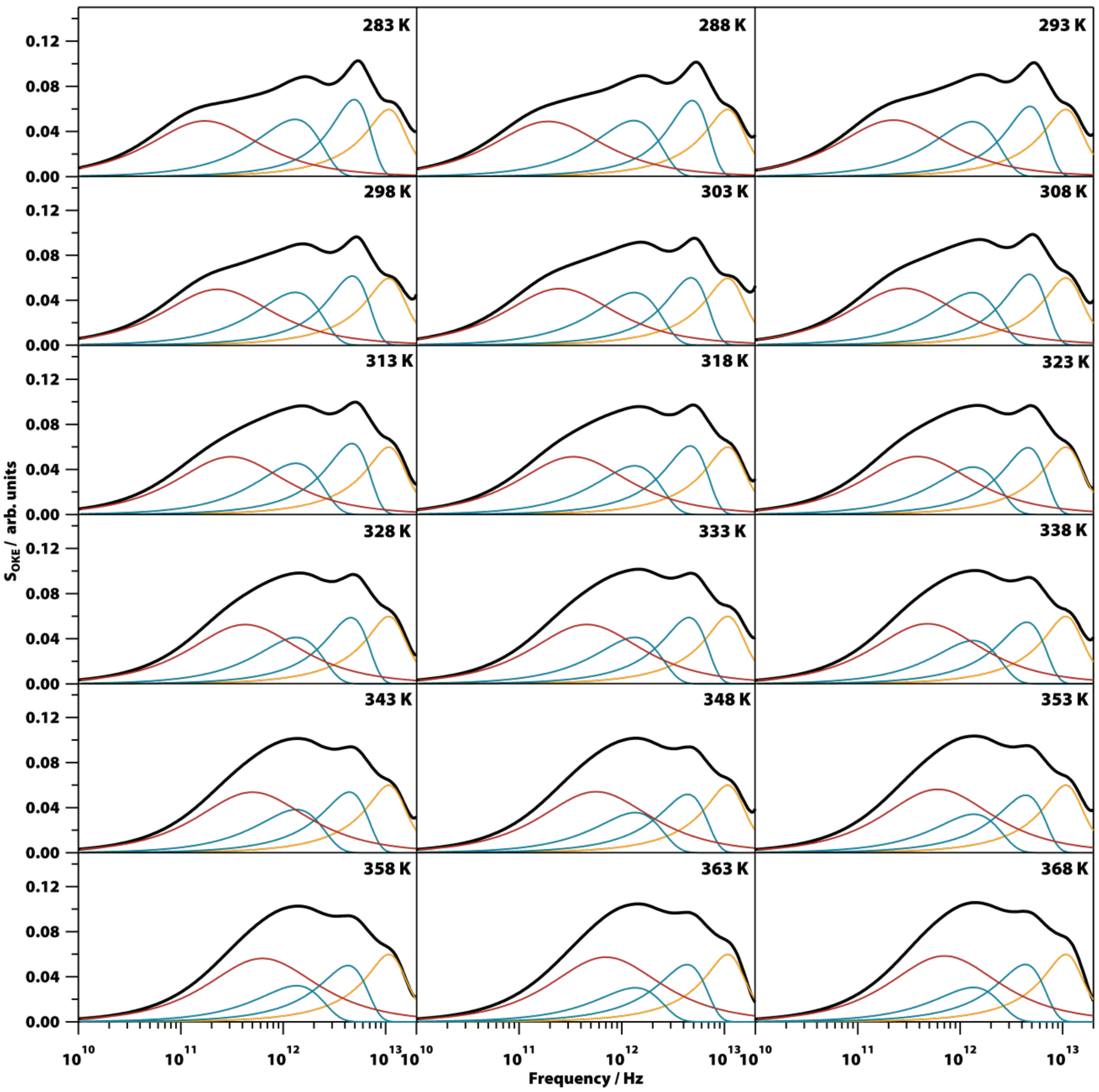

Figure S12. Experimental OKE spectra and the component fit functions used to fit the data for water. Each spectrum has been fitted using the combination of a Cole-Cole function (red), two Gaussian functions (blue) and one Brownian oscillator (yellow).

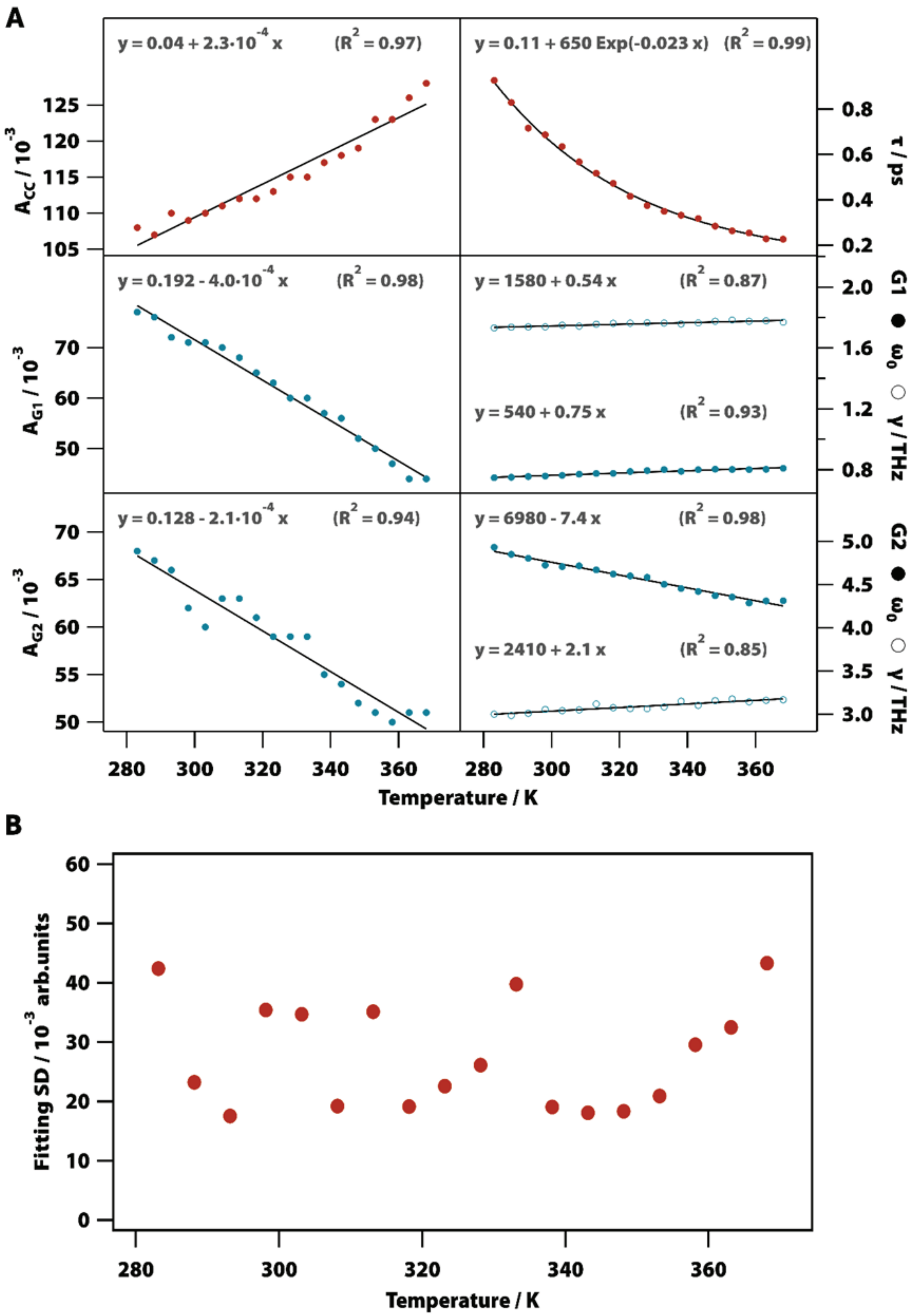

Figure S13. (A) Influence of temperature on the fitted parameters of the functions used to model the water spectra. (B) Standard deviation of the model from the measured OKE spectra of water at each temperature.

2. Supplementary tables

	BA	BB				
**AMP**	1.12 (0.85)	2.37 (1.25)				
**CMP** (1)	1.48 (1.08)	1.58 (1.06)				
**TMP**	1.26 (0.84)	2.41 (1.16)				
**GMP (Li^+/Na^+/K^+/Cs^+)**	0.94 (0.98)	2.15 (1.49)				
	**B1**	**B2**	**B3**	**B4**	**B5**	**B6**
**GMP Li^+ (G4)**	0.38 (0.15)	0.83 (0.56)	1.92 (0.50)	2.56 (0.55)	3.43 (0.46)	
**GMP Na^+ (G4)**	0.39 (0.15)	0.83 (0.56)	1.92 (0.49)	2.56 (0.55)	3.43 (0.46)	5.58 (0.39)
**GMP K^+ (G4)**	0.37 (0.18)	0.87 (0.72)	1.92 (0.50)	2.89 (0.59)	3.45 (0.42)	5.46 (0.39)
**GMP Cs^+ (G4)**	0.32 (0.16)	0.79 (0.56)	1.92 (0.50)	2.56 (0.57)	3.34 (0.51)	5.56 (0.38)
**TBA (G4)**	0.53 (0.17)	1.06 (0.66)	1.87 (0.50)	2.59 (0.63)	3.14 (0.46)	
**d(TG_4T) (G4)**	0.41 (0.20)	0.97 (0.69)	2.04 (0.51)	2.90 (0.63)	3.34 (0.46)	5.57 (0.55)
**d($G_3CT_4G_3C$) (G4)**	0.45 (0.22)	0.93 (0.72)	2.12 (0.56)	2.86 (0.62)	3.44 (0.47)	
	**BC**	**BD**	**BE**	**BF**		
**TBA (ss)**	0.98 (1.27)	1.37 (0.76)	2.13 (0.78)			
**d(TG_4T) (ss)**	0.99 (1.39)	1.39 (0.77)	2.19 (0.67)			
**d($G_3CT_4G_3C$) (ss)**	1.04 (1.27)	1.37 (0.76)	2.13 (0.74)			
**DNA 20mer (ss)**(1)	1.01 (1.39)	1.38 (0.75)	2.19 (0.70)			
**DNA 20mer (ds)**(1)	1.01 (1.39)		2.19 (0.70)	2.83 (0.50)		

Table S1. Fit parameters used to fit the high-frequency part of all the OKE spectra. Frequency ($\omega/2\pi$) and damping rate ($\gamma/2\pi$, in brackets) measured in THz of the Brownian oscillators used to model the vibrational spectra of nucleotides and oligomers with G-quadruplex (G4), single-stranded (ss), and double-stranded (ds) conformations.

Concentration / M	Melting point / K
0.50	270 ± 10
0.60	287.4 ± 0.9
0.70	294.7 ± 0.4
0.80	298.9 ± 0.6
0.90	301.3 ± 0.6
1.00	305.6 ± 0.7
1.10	307 ± 1
1.18	307.5 ± 0.8

Table S2. Melting points for the G4 conformation in Na_2(5′-GMP) at different concentrations obtained for the curves plotted in Supplementary Figure 6.

3. Supplementary Notes

3.1. Supplementary Note 1: Water OKE spectra and subtraction procedure

Measured OKE spectra have contributions from the solvent and the solvated nucleic acids. In order to extract the latter, the spectrum of water was directly subtracted from the measured OKE spectra (Supplementary Figure 11). (2) To simplify the analysis, the spectra of water were simulated in the subtraction process using a model of the dependence of the water spectra on temperature. This model was created after the measurement and fitting of the OKE spectra of water between 283 and 368 K at intervals of 5 K (Supplementary Figure 12). Each spectrum was fitted using a combination of one Cole-Cole function for the diffusive translational motions of water molecules, two gaussian oscillators for the LA and TA phonon modes and one Brownian oscillator for the librational mode at 12.5 THz (Supplementary Table 1). (3, 4) Since this band is scarcely affected by thermal variations in the temperature range of the experiments, (5) the parameters of the Brownian oscillator were kept constant with temperature (ABO = 0.06, $\omega_o/2\pi$ = 12.6 THz, and $\gamma/2\pi$ = 6.8 THz). Supplementary Figure 13-A shows the influence of temperature on each parameter of the employed equations. Only the change on relaxation time with temperature could not be fitted with a linear equation and an exponential fitting had to be used. Most of the fittings show a good regression coefficient. In order to check its quality, the model was compared with the measured spectra and the standard deviation was calculated (Supplementary Figure 13-B). The low values of the standard deviations over the entire temperature range confirm that obtained equations are a good model of the water spectra. This model is a simpler and more accurate update of the model we have previously used. (1) Furthermore, this model comprises a wider temperature range (283 – 368 K).

3.2. Supplementary Note 2: G4s melting thermodynamics

Considering that G4 structures, when denaturize, become GMP molecules, without intermediate states:

$$Q \rightleftharpoons 4A$$

We can estimate the enthalpy and entropy change during the process using van 't Hoff equation for the equilibrium constant:

$$K = \frac{[A]^4}{[Q]}$$
$$\Delta G^\circ = -RT \ln K$$

Substituting, we obtain:

$$\Delta G^\circ = -RT \ln \frac{[A]^4}{[Q]}$$

Formally, the denaturation temperature, or melting point (T_m) is defined as the temperature at which half of the G4 structure is present as free GMP. This is:

$$\frac{[\mathrm{Q}]_0}{2} = [\mathrm{Q}] = 4[\mathrm{A}]$$

Replacing at van 't Hoff equation:

$$\Delta G^\circ = -3\, RT_\mathrm{m} \ln \frac{[\mathrm{Q}]_0}{2^{11/3}}$$

With this equation we can use the data from Supplementary Table 1 to calculate the thermodynamical parameters:

$$\frac{1}{T_\mathrm{m}} = -\frac{3R}{\Delta H^\circ} \ln \frac{[\mathrm{Q}]_0}{2^{\frac{11}{3}}} + \frac{\Delta S^\circ}{\Delta H^\circ}$$

From the regression line in Supplementary Figure 5 we obtain that:

$$\frac{3R}{\Delta H^\circ} = 3.38 \cdot 10^{-4} \pm 2.98 \cdot 10^{-5} \Rightarrow$$
$$\Delta H^\circ = 73 \pm 6 \text{ kJ/mol}$$

and:

$$\frac{\Delta S^\circ}{\Delta H^\circ} = 2.424 \cdot 10^{-3} \pm 7.9 \cdot 10^{-5} \Rightarrow$$
$$\Delta S^\circ = 180 \pm 20 \text{ J/K mol}$$

3.3. Supplementary Note 3: Computational details

All computations were carried out in Gaussian 09 program. (6) PBE0 density functional (7, 8) with Grimme dispersion correction (9) combined with def2-SVP basis set (10) was used as a default method. PBE0 is a hybrid functional shown to provide a satisfactory description for spectroscopic properties (8), while def2-SVP basis set usually allows obtaining adequate estimations for geometry parameters for relatively large molecules consisting of the first and second-row elements in a substantially small CPU time. To check the structural accuracy of PBE0/def2-SVP, M06-2X (11) calculations in def2-TZVP (10) basis set were carried out. The solvent effects were accounted for by using the polarizable continuum model (12) (IEFPCM as implemented in Gaussian) with the dielectric constant $\varepsilon_{static} = 78$ (water).

We do not aim to quantitatively reproduce OKE spectra in this work; instead, we aim to provide an insight into what type of nuclear motions could be responsible for high-intensity signals in the low-frequency part of Raman and OKE spectra. In order to do that, we constructed 1-layer, 2-layer and 3-layer models for G4 complexes with sodium and potassium (see Figure S18). For complexes with potassium, the cation was placed in-between the layers, while for complexes with sodium, the cation was in the plane of the G4 layer. Initial geometry optimizations were performed without symmetry; resulting structures were found to be close to S_8 (2-layer systems), C_4 (3-layer system with Na^+) and C_{4h} (3-layer system with K^+) point groups and symmetrized using Chemcraft software (12) and optimized with symmetry constraints. Harmonic analysis on these structures showed that all Hessian eigenvalues are positive (*i.e.*, there are no imaginary frequencies). The 3-layer model is arguably the smallest reasonable model to show most types of nuclear motion patterns expected in larger stacks (it has two "outer" and one "inner" layer). The majority of calculations was done in vacuum; in order to estimate the solvent effects on calculated spectra, PCM calculations for 2-layer systems were performed.

The OKE intensity for mode *i* was estimated as follows:

$$I_i^{\mathrm{OKE}} \sim \frac{\gamma_i^2}{\tilde{\nu}_i} = \frac{15\,\rho_i\,S_i}{(1+\rho_i)\,\tilde{\nu}_i}$$

where γ_i^2 is an anisotropic polarizability derivative along the mode *i*, and $\tilde{\nu}_i$ stands for the mode wavenumber, ρ_i is a depolarization ratio and S_i is a Raman activity. Spectra were broadened with gaussians with 15 cm^{-1} half-width.

3.3.1. Structure

From the analysis of Table S3 and Table S4 one can see that the equilibrium geometry parameters of the guanine molecule obtained by PBE0-D3/def2-SVP and M06-2X/def2-TZVP are very similar. Moreover, changes in intramolecular distances and bond angles when comparing free guanine and G-quartet without cation are consistent for these two methods. Among the most notable structural changes for the guanine unit is the significant increase in the CO distance coupled with the decrease in adjacent CN distance, resembling a keto-enol tautomerism resulting from a rearrangement of the electron density in the vicinity of the oxygen atom (Figure S15, the equilibrium is shifted to **II** in complexes). Thus, these geometry parameters could serve as local structural descriptors providing an additional argument to distinguish free guanine from that in complexes, *e.g.*, in XRD data.

Non-bonding interactions in the G-quartet without the cation seem to be more difficult to reproduce: PBE0 with def2-SVP and def2-TZVP as well as M06-2X with def2-TZVP show higher discrepancies in hydrogen bond angles and distances. Moreover, while M06-2X provides a planar C_{4h} structure for the G-quartet, a non-planar S_4 equilibrium geometry is obtained by PBE0-D3 both in def2-SVP and def2-TZVP basis sets. Looking at the H-N hydrogen bond length (Table S3 and Table S4), one could speculate that for def2-TZVP structures, the "outer" circle of hydrogen bonds (*i.e.*, H16···N2′) does not exist, unlike for def2-SVP (Figure S16). This is indicated by notably larger distances (r((N6)H16···N2′) is about 2.5 Å while the typical H···Y distance is 1.6–2.0 Å). Hence, among the three different methods used – PBE0-D3 with def2-SVP and def2-TZVP and M06-2X with def2-TZVP – each provides a different geometry, indicating that not only the DFT functional but also the basis set could lead to notably different structures. However, the energy difference between the C_{4h} transition state in PBE0-D3/def2-TZVP (very similar to the minimum in M06-2X) and the nonplanar S_4 minimum

predicted by the same method is only 0.13 kcal/mol, which, given the accuracy of DFT methods, is essentially zero. Apparently, the potential energy surface section along the corresponding intermolecular mode is very shallow (*i.e.*, large changes in geometry are accompanied by small changes in energy), and in that case one should look at the wave function (or probability density) of (at least) the zero vibrational level to resolve the planarity problem (12). Thus, to provide a better understanding of the intermolecular interactions in the G-quartet without a cation, a benchmark study would be desirable, which however goes beyond the scope of this paper.

The structure of the G-quartet with cation is much less sensitive and generally depends on the cation (Figure S17). One can see that the G-quartet with K^+ has a conical shape while with Na^+, the structure is very close to planar. These calculations already indicate where the cations are located in extended G-quadruplexes: K^+ is in-between layers, while Na^+ lies approximately in the layer (in agreement with experiment). (13) Since DFT functionals normally describe the interactions with a cation in a reasonable and robust way, we believe that PBE0-D3/def2-SVP can provide a qualitatively correct picture of the structure and vibrational properties of the G4 complexes with cations. Comparing hydrogen bond distances, one can see that they are quite different in G-quartets with and without cation, hinting at the governing role of cation in the layer geometry (*i.e.*, relative positions of guanines within a layer).

Analysing changes in structural parameters for G-quadruplexes with alkali cations, it should be mentioned that the hydrogen bond distances for different layers are also different; quite expectedly, in 2-layer complexes, they are closer to the "outer" layers in 3-layer complexes. One can also note, along with changes in hydrogen bonds geometry, changes in CO distance: the latter is larger in the outer layers of 3-layer structures, and very similar to that in "free" G4 in the inner layer. Average interlayer distances without cations in Å (and distances between O4 planes, that could be thought of "minimal" interlayer distances) are as follows: 3.22 (2.95); 3.23 (3.07); 3.26 (3.04); 3.24, 3.25 (2.99, 3.06) for 2- and 3-layer complexes with K^+ and Na^+, respectively (two values are given for 3-layer complex with Na^+ because of lower symmetry). These values should correspond to distances between cations in a sufficiently long stack; for our systems, K–K distance is 3.46 Å (from 3-layer complex), and Na–Na are equal to 3.34 (2-layer), and 3.30 and 3.02 Å (in 3-layer system). The calculated K–K distance is in good agreement with the reported experimental value for the 4-layer complex formed in DNA (3.5 Å) (14). For 3-layer complexes, it is to be expected that, due to guanine molecules being non-equivalent in outer and inner layers, notable splittings in vibrational spectra may occur.

3.3.2. Vibrational spectra

Indeed, one can notice that with adding a layer, low-frequency (LF) part of OKE spectra becomes more complicated (Figure S19, Figure S21, Figure S23). A tentative assignment based on visual inspection of the vibrational motions reveals (see Table S9, Table S10, Table S11, and Table S12) that the Raman active modes can be divided into three groups:

1) < 40 cm^{-1} – interlayer modes (dispersion interaction between layers)
2) <150 cm^{-1} (excluding the previous group) – modes involving various displacements of guanine molecules as a whole near their respective equilibrium positions; these could also be described as collective hydrogen bond stretching / bending vibrations.
3) > 150 cm^{-1} - collective intramolecular modes could be approximately described as linear combinations of normal modes of a free guanine molecule.

Modes belonging to the first two groups are purely intermolecular; as one can see, they clearly dominate in LF part for complexes with potassium, to a lesser extent – with sodium. For the first group, there is just one (degenerate) mode that could be easily described as an interlayer displacement. According to our calculations, this mode is present (in this context it means it shows a strong relative intensity) in all studied systems while having the highest intensity in all but 3-layer complex with sodium. Comparing 2- and 3-layer structures, it could be noted that corresponding harmonic frequency decreases (on 10 cm^{-1}); also, it is notably lower in complexes with sodium. From a theoretical viewpoint, this kind of motion is important since it could lead to instability of a large enough stack of guanine layers (supposing there are no other constraints preserving the structure, e.g. side chains in nucleic acid). Indeed, calculations performed for a 2-layer stack with sodium in the PCM model with water (utilizing C_4 symmetry constraint) resulted in a large imaginary

frequency corresponding to this kind of motion. Conversely, the 2-layer complex with potassium optimized within the same procedure resulted in dynamically stable structure that is very similar to that in vacuum.

It is peculiar that intralayer "breathing" mode has shown a notable (still weak) signal only for the 2-layer complex with sodium at approximately 85 cm^{-1}.

There are quite a few modes falling into the second group with their number increasing while adding layers. They are for the most part similar, and the main difference arises from how the amplitudes of corresponding displacements are redistributed within and in between layers. Usually, they are delocalized for the whole structure; however, due to notable structural discrepancy between inner and outer layers, say, modes 17 and 19 in 3-layer complex with potassium are "layer-specific". One could speculate that the increase of the number of layers will lead to less structural deviation and hence, more delocalization for these types of nuclear motions. Having in mind the above discussion of the accuracy (regarding the description of hydrogen bonds) of the method we utilize we do not think that a more sophisticated assignment would be meaningful.

While it is not in the focus of this work, it might also be appropriate to briefly discuss types of nuclear motions leading to higher intensity peaks in the low-frequency part of IR spectra. Bands peaking at approximately 140 and 250 cm^{-1} for all studied G-quadruplexes could be assigned to (*ungerade*) collective G-G ipb and v_3 vibrations and are similar (albeit with a different symmetry) to those observed in OKE spectra. The lowest frequency vibration with notable IR intensity could be tentatively described as Tz mode for cations and their local surrounding and appears in 30-40 cm^{-1} region for complexes with K^+ and 50-60 cm^{-1} for complexes with Na^+. Finally, a mode representing nuclear pattern that does not give rise to notable Raman activity, is a degenerate T_x, T_y mode for cations *only*. *I.e.*, cation "string" is displaced relative to layers. This mode is interesting for not only being (almost uniquely for these systems) well localized but also because its position strongly depends on the cation: it is about 180 cm-1 for both complexes with K^+, 258 cm^{-1} for 2- and 232 cm^{-1} for 3-layer complex with Na^+. It is important to understand that relative intensities of low-frequency (< 350 cm^{-1}) to high-frequency part for IR spectra are very small, so all discussed IR features may be very difficult to verify experimentally.

While it is possible to provide an intuitive explanation upon relative intensities in IR spectra, rationalizing Raman (or OKE) spectra is a less straightforward problem. In order to provide at least basic considerations, one can look at the symmetry of corresponding modes. In dipole approximation, fundamental transitions belonging to the following representations are allowed in Raman:

1) S_8 point group (2-layer systems): A, E_2, E_3.
2) C_{4h} (3-layer with K^+): A_g, B_g, E_g.
3) C_4 (3-layer with Na^+): A, B, E (all).

Analysing Table S9, Table S10, Table S11, and Table S12, it is evident that for all complexes normal modes belonging to degenerate representations (E_3, E_g, E) lead to higher relative intensities in the Raman spectrum. However, many strong signals in the computed spectrum of the 2-layer complex with Na^+ belong to a fully symmetric (A) representation. Thus, it is easy to show that most Raman-active modes have a rotation symmetry: (R_x, R_y) transform as E_3, E_g, E in S_8, C_{4h}, and C_4 point groups, respectively, while R_z transforms according to A (or A_g) irreducible representation. The fact why the 2-layer complex with sodium shows somewhat different behaviour remains unexplained.

Finally, comparing calculated OKE spectra for G4 complexes in vacuum and in polarized media (Figure S19 and Figure S23) one can see that solvent effects should definitely be accounted for when aiming to reproduce the intensity pattern in LF part, *e.g.*, the relative intensity of interlayer displacement mode is smaller in water than in vacuum. However, structure, vibrational patterns and harmonic frequencies are very similar for these two complexes; thus, we believe that our qualitative conclusions would in general be supported by future in-depth studies.

In order to provide a more rigorous, quantitative theoretical description for the systems in question, one should 1) use a better method (def2-SVP basis set is too small to provide a good description of a hydrogen bond and polarizability); 2) investigate larger stacks (probably using PBC calculations, or better, modelling finite stacks with a number of layers close to observed in our experiment); 3) go beyond double harmonic approximation when it comes to spectra.

3.3.3. Structure of guanine complexes

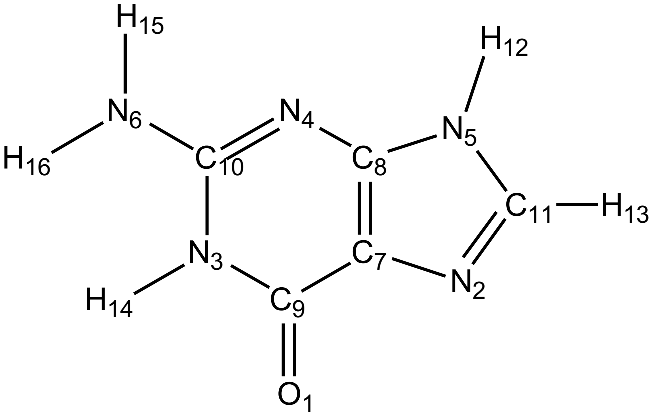

Figure S14. Atomic enumeration in guanine fragment.

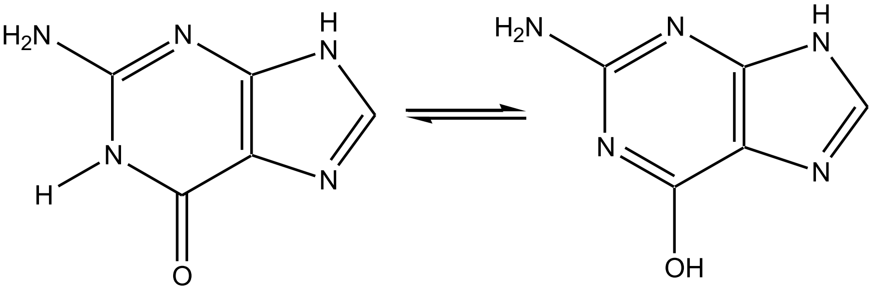

I

II

Figure S15. Two tautomeric forms of guanine molecule.
